# Supplementary material for: Content-rich biological network constructed by mining PubMed abstracts
Source: BMC Bioinformatics. 2004 Oct 8;5:147. doi: 10.1186/1471-2105-5-147 (PMC528731; doi:10.1186/1471-2105-5-147)
Supplement: Additional File 2 — The original results of the above study (non-essential files are deleted to keep the file size under the limit set by BMC bioinformatics). [file 1471-2105-5-147-S2.bz2 › chilibotAdditionalFile2/dip05/102ID7937747E432/html/MCL1_BCL2.html]

 


 **MCL1** and **BCL2** 
  
Found 328 abstracts in PubMed, retrieved 05.  
 

 What does Google say? 
 PDF only 
| .edu only 

---

**Interactive relationship** (e.g. stimulation, inhibition, etc)

**Neutral relationship**- Here we show that Bid, a crucial proapoptotic protein that integrates the action of other Bcl 2  [ **BCL2** ]  family members, exhibits discrete specificity for metabolites of cardiolipin, especially monolysocardiolipin MCL  [ **MCL1** ] .  Ref: 12894218 Cell Death DifferCell Death Differ,
- MCL  [ **MCL1** ]  may thus play a crucial role in connecting lipid metabolism, relocation of Bid to mitochondria and integrated action of Bcl 2  [ **BCL2** ]  proteins on mitochondrial membranes.  Ref: 12894218 Cell Death DifferCell Death Differ,

**Non-interactive relationship** (e.g. studied together, co-existance, homology, etc.)

- In contrast, treatment of cells with LY294002, to inhibit phosphoinositide 3 kinase PI3K, caused downregulation of Bcl 2  [ **BCL2** ]  and Mcl 1  [ **MCL1** ]  and allowed DeltaMEKK1 ER\* to elicit a robust apoptotic response characterized by activation of Bax and caspases.  Ref: 12879014 Oncogene, 2003
- Expression of the anti apoptosis proteins, Bcl 2  [ **BCL2** ]  and Mcl 1  [ **MCL1** ]  expression decreased time dependently while Bax expression increased.  Ref: 12883267 Pancreas, 2003
- The stress induced transcription factor GADD153 and the  **Bcl2**  related  [ **MCL1** ]  protein Bak are upregulated in response to fenretinide.  Ref: 12880976 Cancer Lett, 2003
